# Deletion of atypical type II restriction genes in *Clostridium cellulovorans* using a Cas9-based gene editing system

**DOI:** 10.1007/s00253-025-13404-6

**Published:** 2025-01-29

**Authors:** Aline I. Schöllkopf, Luciana Almeida, Karina Krammer, Cristina González Rivero, Wolfgang Liebl, Armin Ehrenreich

**Affiliations:** 1 Chair of Microbiology, Technical University of Munich, TUM School of Life Science, Emil-Ramann-Str. 4, 85354 Freising, Germany; 2 Current address: Max Von Pettenkofer-Institut, Pettenkoferstraße 9A, Munich, Germany; 3 Current address: Bayerische Landesanstalt Für Landwirtschaft, Lange Point 4, Freising, Germany

**Keywords:** *Clostridium cellulovorans*, CRISPR/Cas9, Promoter strength, Riboswitch, Type II restriction-modification system, Conjugation

## Abstract

**Abstract:**

The anaerobic bacterium *Clostridium cellulovorans* is a promising candidate for the sustainable production of biofuels and platform chemicals due to its cellulolytic properties. However, the genomic engineering of the species is hampered because of its poor genetic accessibility and the lack of genetic tools. To overcome this limitation, a protocol for triparental conjugation was established that enables the reliable transfer of vectors for markerless chromosomal modification into *C. cellulovorans*. The availability of reporter genes is another requirement for strain engineering and biotechnological applications. In this work, the oxygen-free fluorescence absorption-shift tag (FAST) system was used to characterize promoter strength in *C. cellulovorans*. Selected promoters were used to establish a CRISPR/Cas system for markerless chromosomal modifications. For stringent control of expression of Cas9, a theophylline-dependent riboswitch was used, and additionally, the anti-CRISPR protein AcrIIA4 was used to reduce the basal activity of the Cas9 in the off-state of the riboswitch. Finally, the newly established CRISPR/Cas system was used for the markerless deletion of the genes encoding two restriction endonucleases of a type II restriction-modification (RS) system from the chromosome of *C. cellulovorans*. In comparison to the WT, the conjugation efficiency when using the deletion mutant as the recipient strain was improved by about one order of magnitude, without the need for prior *C. cellulovorans*-specific in vivo methylation of the conjugative plasmid in the *E. coli* donor strain.

**Key points:**

*• Quantification of heterologous promoters enables rational choice for genetic engineering.*

*• CRISPR/Cas with riboswitch and anti-CRISPR allows efficient gene deletion in C. cellulovorans.*

*• Conjugation protocol and type II REase deletion enhance genetic accessibility.*

## Introduction

The anaerobe *Clostridium cellulovorans* is a promising candidate for the sustainable production of biofuels and platform chemicals. Due to its cellulosome, a multi-enzyme complex of cellulases, *C. cellulovorans* can utilize crystalline cellulose without pre-treatment (Sleat et al. 1984; Shoseyov and Doi 1990; Tamaru et al. 2010).

However, genome editing of the strain is hampered because of its poor genetic accessibility and the lack of suitable genetic tools (Yang et al. 2015; Bao et al. 2019b). The introduction of recombinant DNA, such as plasmids allowing genetic modifications, is often impeded by restriction-modification (RM) systems present in the host bacteria (Mermelstein and Papoutsakis 1993; Jennert et al. 2000). The deletion of the endonucleases of RM systems can increase the transformation efficiency dramatically in other Clostridia (Lesiak et al. 2014; Huang et al. 2018). In *C. cellulovorans*, previous reports showed that a putative type II RM system is likely the most relevant restriction system. This type II RM system is unusual because it is encoded by a gene cluster containing ORFs for two putative restriction endonucleases, Clocel_4005 and Clocel_4006, and two putative methyltransferases, Clocel_4007 and Clocel_4008 (Yang et al. 2016).

The transformation of C*. cellulovorans* with plasmid DNA was reported for in vivo methylated plasmids and for plasmids where the predicted restriction sites of the type II system were avoided, with only low efficiency for both approaches (Yang et al. 2015; Bao et al. 2019b, 2019a, 2021). On the other hand, the effect of a chromosomal deletion of the type II RM system was not yet described because suitable tools for markerless modifications for *C. cellulovorans* were not available.

In recent years, the number of tools for the genetic engineering of Clostridia has dramatically increased, including the utilization of clustered regularly interspaced short palindromic repeats/CRISPR-associated proteins 9 (CRISPR/Cas9) systems (Minton et al. 2016; Joseph et al. 2018). Within this system, desired homologous recombination events are selected after a directed DNA strand break caused by the sequence-specific guided nuclease Cas9. The frequency of homologous recombination leading to the desired mutation should be very high due to the lethality of double-strand breaks (DSB) in many prokaryotes. Thus, proper control of the nuclease activity with regard to expression level and time point is important to circumvent adverse effects on transformation and off-target cuts of the chromosome. Recently, various methods have been described to modulate the Cas9 activity in bacteria, involving the control of the Cas9 expression by inducible promoters or reducing the activity by the co-expression of anti-CRISPR proteins (Cañadas et al. 2019; Wasels et al. 2020; Stukenberg et al. 2022). Both methods require promoters of known strength to orchestrate the level of protein expression. The application of the fluorescence absorption-shift tag (FAST) system (Plamont et al. 2016) was described as a reporter system for different anaerobes because in contrast to systems relying on green fluorescent protein (GFP) and derivatives thereof it works in the absence of oxygen and provides a strong fluorescence signal (Streett et al. 2019; Charubin et al. 2020; Flaiz et al. 2021, 2022; Hernandez and Costa 2022; Hocq et al. 2023).

The work reported here served to develop a protocol for the reliable transconjugation of DNA from *E. coli* to *C. cellulovorans* 743B and to establish a Cas9-based chromosome editing system for *C. cellulovorans*. To achieve this, we decided to also establish a FAST reporter system for the characterization of different heterologous promoters with regard to their strength in *C. cellulovorans*. The data obtained were to be used for rational promoter selection and construction of a deletion vector containing *cas9* under the control of a theophylline-responding riboswitch (Topp et al. 2010; Cañadas et al. 2019) and to attempt further reduction of the basal Cas9 activity by the anti-CRISPR protein AcrIIA4 (Rauch et al. 2017). Finally, to prove the utility of this new Cas9-based chromosome editing system for *C. cellulovorans* and simultaneously engineer an improved *C. cellulovorans* strain for future genetic modification approaches, we targeted the predicted type II restriction endonucleases Clocel_4005 and Clocel_4006 for deletion from the chromosome of *C. cellulovorans*.

## Materials and methods

All strains used in this study are listed in Table 1. *E. coli* NEB10B was used to propagate plasmids and as a donor for transconjugation experiments, while *E. coli* CA434 was used as a helper strain for triparental conjugation. *E. coli* strains were grown aerobically with orbital shaking (180 rpm) at 37 °C in liquid LB medium or on solid LB agar (1.5% (w/v)). Appropriate antibiotics were added at the following concentrations: tetracycline, 10 µg/mL, and chloramphenicol, 34 µg/mL.

**Table 1:** List of strains used in this study

Stain	Characteristics	Source
*Clostridium cellulovorans* 743B	WT	DSM3052
*Clostridium cellulovorans* ΔtypII	ΔClocel_4005, ΔClocel_4006	This study
*Clostridium sporogenes* strain DSM 795	WT	DSMZ
*Clostridium acetobutylicum* ATCC 824	WT	DSMZ
*Clostridium saccharobutylicum* DSM 13864	WT	DSMZ
*Clostridium ljungdahlii* DSM 13528	WT	DSMZ
*Escherichia coli* NEB10B	*Δ(ara-leu) 7697 araD139 fhuA ΔlacX74 galK16 galE15 e14-ϕ80dlacZΔM15 recA1 relA1 endA1 nupG rpsL (Str*^*R*^*) rph spoT1 Δ(mrr-hsdRMS-mcrBC)*	NEB
*E. coli* CA434	R702 plasmid	Des Purdy et al. (2002)

*C. cellulovorans* 743B was purchased from DSMZ (DSMZ-Deutsche Sammlung von Mikroorganismen und Zellkulturen GmbH, Braunschweig, Germany). The growth medium was adapted from DSMZ’s 520 medium ((NH_4_)_2_SO_4_, 1.3 g/L; KH_2_PO_4_, 1.5 g/L; K_2_HPO_4_, 2.9 g/L; FeSO_4_ × 7 H_2_O solution (0.1% (w/v) in 0.1 N H_2_SO_4_), 1.25 mL/L; SL10, 1 mL/L; Na-resazurin solution (0.1% (w/v), 0.5 mL/L; MgCl_2_ × 6 H_2_O, 980 µM; CaCl_2_ × 2 H_2_O, 510 µM; Na_2_CO_3_, 14 mM; l-cysteine HCl × H_2_O, 0.5 g/L) and supplemented with 0.2% (w/v) yeast extract (2YE) or tryptone (2 T); 0.5% (w/v) carbon source; 0 mM, 10 mM, or 100 mM HEPES buffer (pH 7.0). A 1% (w/v) agar was added for solid media. The cells were cultivated statically under anaerobic conditions at 37 °C. For long-time storage, the strain was supplemented with 25% glycerol (v/v) and stored at − 80 °C.

Cell growth was controlled via optical density by OD_600_ or McFarland (McF) densitometer in McF units (DEN-1B Densitometer; Grant Instruments, Royston, United Kingdom).

### Synthetic constructs

#### RS

TTTTTATCAGGAAACAGCTATGACCGCGGCCGCTACAGTTATATAAAAATTACTTTAAAAATTAATAAAAACATGGTAAAATATAAATCGGTACCAATACGACTCACTATAGGTTCCGGTGATACCAGCATCGTCTTGATGCCCTTGGCAGCACCCTGCTAAGGAGGCAACCATATGGAACACGTAGCATTTGGAAGTGA

#### acrIIA4 cassette

CCTGCAGGATAAAAAAATTGTAGATAAATTTTATAAAATAGTTTTATCTACAATTTTTTTATCAGGAAACAGCTATGACCGCGGCCGCATTCAGTTCAGATTTTAATGTAATAGTCTGCATATCATTGTAAAACTCCTCTTCATCTTCATACTCTTGGTTCCAGCCATTCTTGAACGCGCTTATAAATTTTTCGACAATCGATTCGTTTTCGCTTTCGCTGATCACGTATTCATTTCCATCATTGTTAACGCGGATAATAAGCTGGGTAATTGAATTGGAATCGGTACCACTCAATTTTACCGTATAATCCTTGTTTTTGATCTCACGAATCAGGTCGTTAATGTTCATATGTATTCCTCCTATTTTTCATTTCATAAG.

#### sgRNA cassette

CCGTATTACCGCCTTTGAGTTATCATTCTGTGGGAAGCCATTTTCTGGCTTCCCTATTTTTAAACTGACGTCCCCGGGACTAGTTTTATATTTAGTCCCTTGCCTTGCCTACAAGGGATTTCCTATTCCTTTCATTTACAATTCATACGTATAAAATCCAAATTTTTCTTGACATTTATACACATAAATATTATGATTTATATAGGTAATCGCTTTCATAAAATATATTACCCTTAGGAAATCAAATGATTATAAGTCATATATGAAAACGTTATATATAATTGATATGTTTACATTTGTAACTTAGATTTCTCTTTGATTTCCACATATATAAATCTTAAGGAGGAGTTTTCGTCGACGAAACTTGACTGGTACAGGTGTTTTAGAGCTAGAAATAGCAAGTTAAAATAAGGCTAGTCCGTTATCAACTTGAAAAAGTGGCACCGAGTCGGTGCTTTTTTTGAATTCCTCGAGACTGTATAAATAAGGCCTTTTATGTTTAATCACATAAAAGGCCTTTAAATATAAGATGGATCTTTTCCGCTGCATAA.

#### DNA techniques and genetic manipulation

Routine molecular biological procedures were performed according to the protocols supplied by the manufacturers of enzymes and kits. PCR fragments were amplified using the Q5 High-Fidelity DNA Polymerase (NEB) for standard reactions, the PrimeSTAR DNA Polymerase (TAKARA BIO INC., Kusatsu, Japan) for large fragments, and the Phire II Hot Start DNA Polymerase (Thermo Fisher Scientific, Waltham, MA, USA) for analytical reactions. The NucleoSpin PCR clean-up gel extraction and the Plasmid EasyPure kit (Macherey–Nagel, Düren, Germany) or Zymoclean Gel DNA Recovery Kits (Zymo Research Europe GmbH, Freiburg, Germany) were used for DNA purification. Plasmids (Table 2) were constructed using restriction-based and SLiCE or NEBuilder isothermal assembly techniques (Zhang et al. 2012; Gibson et al. 2009). DNA modification enzymes were purchased from New England Biolabs (Ipswich, MA, USA) or Thermo Fisher Scientific (Waltham, MA, USA), and oligonucleotides were purchased from Eurofins Genomics Germany GmbH (Ebersberg, Germany) and Sigma*-*Aldrich (Taufkirchen, Deutschland) (Table 3).

**Table 2:** Plasmid used in this study

**Plasmid**		
pACYC184	P15A replicon, tetR, cmR	
pUC19	P(lac), lacZ	
placORMI	P15A, tetR, P(lac), Clocel_4007 & Clocel_4008	This study
pMTL83151	pCB102 replicon, ColE1, oriT, catP, MCS	Heap et al. (2009)
pMTL88151	P19 replicon, ColE1, oriT, catP, MCS	SBRC, Nottingham
pMTL83155	pCB102 replicon, ColE1, oriT catP, FAST gene	SBRC, Nottingham
pMTL83155-Cac-araE	pCB102 replicon, ColE1, oriT catP, FAST gene, ParaE	This study
pMTL83155-Csporo-fdx	pCB102 replicon, ColE1, oriT catP, FAST gene, Pfdx	This study
pMTL83155-Csa-fdx	pCB102 replicon, ColE1, oriT catP, FAST gene, Pfdx	This study
pMTL83155-Clju-upp	pCB102 replicon, ColE1, oriT catP, FAST gene, Pupp	This study
pMTL83155-Csa-thl	pCB102 replicon, ColE1, oriT catP, FAST gene, Pthl	This study
pMTL83155-RS	pCB102 replicon, ColE1, oriT catP, FAST gene, RS	This study
pChN	pBR322 replicon, catP, codBA gene	Huang et al. (2018)
pAS5	pBR322 replicon, catP, codBA gene, repH replicon, *Stu*I site	This study
pAS5-delClocel_4005&4006	pBR322 replicon, catP, codBA gene, repH replicon, 700 bp HR	This study
pAcrCas_delClocel4005&4006_ori3	pBR322 replicon, pCB102 replicon, catP, P_RS_-cas9, P_upp_-acrIIA4, P_araE_-sgRNA	This study
pAcrCas_delClocel4005&4006_ori8	pBR322 replicon, p19 replicon, catP, P_RS_-cas9, P_upp_-acrIIA2, P_araE_-sgRNA, Clocel4005&4006 flanking regions,	This study
pAcrCas	pBR322 replicon, p19 replicon, catP, P_RS_-cas9, P_upp_-acrIIA4, P_araE_-sgRNA, *Eco*32I and *Bsa*I MCS	This study

**Table 3:** Oligonucleotides used in this study

Name	Sequence	Application
**AS13_SacI_4008_rv**	cacGAGCTCGAAATGAGGATAGTTTTATGAGTAAGAAATTTAC	placORMI
**AS14_XhoI_4007-fw**	cacCTCGAGGATACACGGACTACACCTCC	placORMI
**AS15_XhoI_pacyc-rv**	acaCTCGAGATTCTTGGAGTGGTGAATCC	placORMI
**AS16_SacI_pacyc-fw**	cacGAGCTCATCTCGATAACTCAAAAAATACG	placORMI
**AS17_Slice_pACYC_rv**	ctatgcggcatcagagcagATTGTACGTATTTTTTGAGTTATCGAG	placORMI
**AS18_Slice_pACYC_fw**	agtgagctaactcacattaattgcgtGGTGAAAGTTGGAACCTCTTAC	placORMI
**AS19_Slice_pUC_fw**	atctcgataactcaaaaaatacgCTATGCGGCATCAGAGCAG	placORMI
**AS20_Slice_pUC_rv**	cacgtaagaggttccaactttcacCACGCAATTAATGTGAGTTAGC	placORMI
**AS68_NotI_Cljd-upp_fw**	accGCGGCCGCAGACACCAAAGAAGGATAGATAAAATTAC	pMTL83155
**AS69_NdeI_Cljud-upp_rv**	ttcCATATGTATTCCTCCTATTTTTCATTTCATAAGG	pMTL83155
**CGR3_NdeI_Cspo_pfdx_rv**	gtatgcCATATGTAACACACCTCATATGc	pMTL83155
**CGR4_NotI_Cspo_pfdx_fw**	gctaaGCGGCCGCGTGTAGTAGCCTGCGAAATAAG	pMTL83155
**CGR5_NotI_Csac_pfdx_fw**	acatagGCGGCCGCGGGTATATAGTCATTTAATAGTGATTTATAGA	pMTL83155
**CGR7_NdeI_Csac_pfdx_rv**	cgaCATATGGAACACCTCCTAATAAATTGTGG	pMTL83155
**CGR10__NdeI_fdx-E_rv**	GTTCCATATGGTTGCCTCCTTAGCAGG	pMTL83155
**ES1_NotI_P(Cac-araE)_fw**	ataGCGGCCGCTTTATATTTAGTCCCTTGCCTTGCC	pMTL83155
**ES2_NdeI_P(Cac-araE)_rv**	ataCATATGAACTCCTCCTTAAGATTTATATATGTGG	pMTL83155
**BP1_NotI_P(Cac-thl)_fw**	ataGCGGCCGCTTTTTAACAAAATATATTGATAAAAATAATAATAGTGGG	pMTL83155
**BP2_NdeI_P(Cac-thl)_rv**	ataCATATGAACTAACCTCCTAAATTTTGATACGG	pMTL83155
**S29_cas9_fw**	ATGGATAAGAAATACTCAATAGGCTTAGATATCG	*cas9* gene, pAcrCas
**S30_cas9_rv**	CAGTCACCTCCTAGCTGACTC	*cas9* gene
**Del3-Clocel_4004_fw**	GCTTTACAGATAGCCACTACATTTGC	Deletion locus type II RMS
**Del4-Clocel_4008_rv**	GCTGTTCCACCGATGCTAGGGC	Deletion locus type II RMS
**AS74_pChN_fw**	tatgttcccgtctacgtttaaacAAGATCCTTTTTGATAATCTCATGACC	pAcrCas
**AS75_pChN_rv**	atttttatgctagcaggcctCTTATCTTACACACAATTAAGTAGAAGAACC	pAcrCas
**AS76_4004_fw_AB**	attgtgtgtaagataagaggTATCAGCATTGTTCCCGGTTC	pAcrCas
**AS77_4004_rv_A**	tggaggtgtagTAAGAGATTTAAAAGACATTTGTGTGAGG	pAcrCas
**AS78_4007_fw_A**	taaatctcttaCTACACCTCCATTTCTTTTAACAAATC	pAcrCas
**AS79_4007_rv_AC**	cttatttttatgctagcaggCGAAATAATAGATGATTTGCGGATTG	pAcrCas
**ES10_ChN-pRB3200_rv**	aaaatggcttcccacagaatgataACTCAAAGGCGGTAATACGGTTATCC	pAcrCas
**ES11_oriT_fw**	taaatataagatGGATCTTTTCCGCTGCATAACC	pAcrCas
**ES12_oriT_rv**	tacaatttttttatcctgcaggCTTCCTTTCTCGCCACGTTCG	pAcrCas
**AS114_upp-acr_fw**	gttcatatgTATTCCTCCTATTTTTCATTTCATAAGG	pAcrCas
**AS111_upp-RS_rv**	atctacatctacaaaataatcatcatatataacacttacttcttttttttccttacttatttcacaggctactacacGCGGCCGCAGACACCAAAGAAGG	pAcrCas
**AS112_RS-upp_fw**	TGATGATTATTTTGTAGATGTAGATAGGATAATAGAATCCATAGAAAATATAGGTTATACAGTTATATAAAAATTACTTTAAAAATTAATAAAAACATGG	pAcrCas
**AS113_RS-cas9_rv**	gatatctaagcctattgagtatttcttatccatATGGTTGCCTCCTTAGCAGG	pAcrCas
**S29**	ATGGATAAGAAATACTCAATAGGCTTAGATATCG	pAcrCas
**ES17_SpyCas9_rv**	tttaaaaaataataaagcttTCAGTCACCTCCTAGCTGACTC	pAcrCas
**ES13_pAS5-TcodBA_fw**	agctaggaggtgactgaAAGCTTTATTATTTTTTAAATAAATATAGTGTTATTTCAAAAG	pAcrCas
**ES9_pAS5-pRB3200_fw**	gttaccctaagtttaaacAAGATCCTTTTTGATAATCTCATGACC	pAcrCas
**SalI_sgRNA_rv**	GTCGACGAAAACTCCTCCTTAAG	pAcrCas
**SalI_sg2_fw**	ttcGTCGACataatctcattcttttccgtGTTTTAGAGCTAGAAATAGCAAGTTAAAATAAGG	pAcrCas
**AS122_Eco47I_BsaI_Tfdx_fw**	gagaccAGCGCTGGTCTCCTAGCATAAAAATAAGAAGCCTG	pAcrCas
**AS128_BsaI_MCS_rv**	gcgctGGTCTCCTTATCTTACACACAATTAAGTAGAAGAACC	pAcrCas

Restriction enzyme recognition sites introduced in primers for the purpose of plasmid construction are underlined. Nucleotides at the 5**′** end that do not bind to the target site are indicated as small letters

#### Triparental conjugation of *C. cellulovorans*

DNA transfer was done by triparental conjugation using the helper strain *E. coli* CA434 and *E. coli* NEB10B as donors bearing the plasmid of interest and the methylation plasmid as indicated. The *E. coli* strains were inoculated to an OD_600_ of 0.1 in an LB medium supplemented with the appropriate antibiotics and 0.1 mM IPTG, while *C. cellulovorans* was inoculated into 2YE-cellobiose medium with 10% (v/v) of an overnight culture. All strains were grown to exponential phase (McF value of 2.5 for *C. cellulovorans* and McF value of 3.5 for *E. coli*). One mL each of helper and donor *E. coli* were mixed and washed two times with phosphate-buffered saline (PBS, pH 7.4), and the pellet was resuspended anaerobically in 250 µL *C. cellulovorans* culture (ca. 10^5^ recipient cells/mL). The mixture was spotted in six spots of 40 µL each onto plates containing 0.2% (w/v) yeast extract, 0.5% (w/v) glucose, and 100 mM HEPES (pH 7.0). After 20–24 h of incubation at 37 °C, the cell mass was resuspended in PBS and plated on selection plates (2 T medium) containing 0.2% (w/v) tryptone, 0.5% (w/v) cellobiose, 10 mM HEPES, 50 µg/mL d-cycloserine, and 10 µg/mL thiamphenicol and incubated anaerobically for 1–3 days at 37 °C. The conjugation efficiency was calculated as thiamphenicol-resistant transconjugants per recipient cell.

#### Identification of mutants and plasmid curing

*C. cellulovorans* transconjugants were cultivated overnight in 2 T cellobiose medium supplemented with 10 µg/mL thiamphenicol (2T_Thia_). Samples from a serial dilution were spread on induction plates containing 0, 2.5, and 5 mM theophylline and 10 µg/mL thiamphenicol. The genotype of clones obtained was analyzed by amplifying the chromosomal target region via PCR with Phire Green Hot Start II DNA polymerase from the total DNA or colony biomass, and the identity of mutants was confirmed by Q5 polymerase PCR and sequencing.

Plasmid-cured deletion strains were obtained by cultivating clones with a chromosomal deletion in a liquid medium without antibiotic pressure. For this, plasmid-carrying deletion strains were inoculated into a liquid 2T_Thia_ medium and subsequently cultivated in an antibiotic-free 2 T medium. Dilution streaks of the cultures were done on 2 T plates. Single colonies were streaked on 2 T plates with and without 10 µg/mL thiamphenicol to identify thiamphenicol-sensitive clones. The plasmid loss of antibiotic-sensitive colonies was verified by PCR analysis of the presence of the *cas9* gene (primers S29 and S30). Additionally, the colony was inoculated into liquid 2 T medium with and without thiamphenicol to check for antibiotic sensitivity.

#### FAST reporter

The cells were grown in 2 T or LB medium supplemented with appropriate antibiotic and inducer and were harvested in the mid or stationary phase as indicated. The cells were washed two times in PBS, mixed with an equal amount of a 10 µM ^TF^Lime fluorophore solution (The Twinkle Factory, Paris, France), and measured in a black 96-well plate (#655900; Greiner AG, Kremsmünster, Österreich) with an excitation wavelength of 480 nm and emission wavelength of 530 nm in technical triplicates in a SPECTROstar Omega microplate reader (BMG Labtech, Ortenberg, Germany). OD_600_ was determined to normalize the fluorescence intensity to the cell density (FLU/OD_600_), and data were presented relative to the negative control to account for background fluorescence of the cells bearing the vector control pMTL83155 (Fold Change negative, FC_neg_). Data analysis was performed using GraphPad Prism Version 7.0 software. The significance level was calculated with an unpaired two-tailed student’s *t*-test with Welch’s correction.

## Results

### Development of a protocol for triparental conjugation of *C. cellulovorans*

**Fig. 1:**
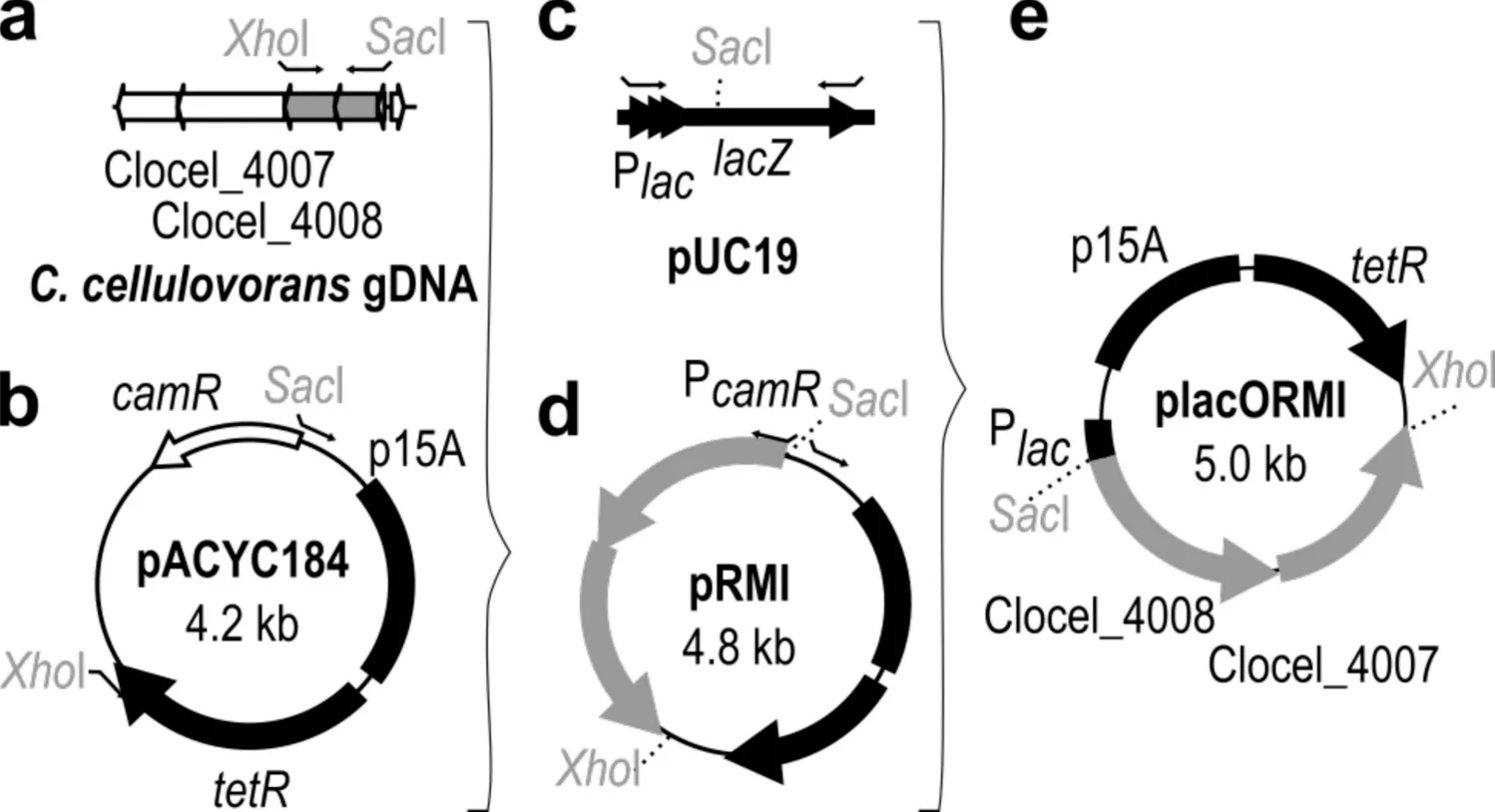
Schematic construction of the in vivo methylation vector placORMI. **a** Genes of type II RM system operon on the genome of *C. cellulovorans* containing the methyltransferases Clocel_4007 and Clocel_4008; **b** pACYC184 vector containing the Gram-negative replicon p15A and the tetracycline resistance cassette tetR; **c** pUC19 vector containing the *lac* operon; **d** pRMI vector containing the methyltransferases Clocel_4007 and Clocel_4008 under the control of the *camR* promoter of pACYC184; **e** placORMI, a methylation plasmid for *C. cellulovorans* with the methyltransferases Clocel_4007 and Clocel_4008 under the control of the inducible lac promoter of pUC19, the Gram-negative replicon p15A and the tetracycline resistance cassette *tetR* of pACYC184. The primer binding sites are marked

In order to improve the limited genetic accessibility of *C. cellulovorans* WT strains, a protocol for triparental conjugation was developed that allows the transfer of various reporter or deletion plasmids in *C. cellulovorans*. First, however, it was important to address the problem that restriction-modification (RM) systems pose an important barrier to genetic engineering in Clostridia (Lesiak et al. 2014; Huang et al. 2018). Building upon previous studies that identified a type II RM system as a predominant restriction barrier in *C. cellulovorans* (Yang et al. 2016), we constructed an in vivo methylation plasmid placORMI containing the ORF for methyltransferases Clocel_4008 and Clocel_4007 under the control of an inducible *lac* promoter to overcome the host’s defense system (Fig. 1).

To this end, the ORFs encoding methyltransferases Clocel_4008 and Clocel_4007 were amplified from genomic DNA using the primers AS13 and AS14 (all primers used in this study are listed in Table 3) and inserted into a linearized pACYC184 backbone (primer AS15 and AS16) under the regulatory control of the promoter of the vector’s chloramphenicol resistance marker. To fine-tune expression levels, this methylation plasmid pRMI was linearized (primer AS17 and AS18), and the promoter region was replaced with the *lac* promoter from pUC19 (primer AS19 and AS20) and ligated after restriction with *Sac*I, yielding the methylation vector placORMI.

A protocol for triparental conjugation for *C. cellulovorans* was established by co-transforming the in vivo methylation plasmid placORMI and the shuttle plasmid pMTL83151 (Heap et al. 2009; Yang et al. 2016) into *E. coli* NEB10B followed by conjugation into *C. cellulovorans* strain 743B as described in the “materials and methods” section.

Expression of the methyltransferase genes of the in vivo methylation plasmid placORMI was induced by 0 mM, 0.1 mM, and 1 mM IPTG in *E. coli*, where an optimal inducer concentration of 0.1 mM IPTG was determined. During the mating process, plates were supplemented with 0.5% (w/v) glucose as a carbon source, which provides the required energy for the transconjugation of *C. cellulovorans* and *E. coli*. Glucose showed the best results in comparison to cellobiose- and lactose-containing mating plates because both *E. coli* strains we used can not utilize cellobiose or lactose and the energy supply of the donor was found to be important. As *C. cellulovorans* was described to be sensitive toward low pH values (Sleat et al. 1984), we included 100 mM HEPES in the bicarbonate buffered mating plates to stabilize the pH.

For the selection of *C. cellulovorans* 743B transconjugants, 10 µg/mL thiamphenicol was included in the selection plates, and the counter-selection of *E. coli* donor cells after conjugation was achieved by the inability of *E. coli* to use cellobiose. Yeast extract was replaced by tryptone in the selection plates (2 T medium) to avoid the presence of other fermentable carbohydrates that could have allowed the growth of *E. coli*. As *C. cellulovorans* produces small amounts of glucose during growth on cellobiose (Inamori et al. 2016), the resulting slight background growth of *E. coli* was repressed by adding d-cycloserine (final concentration of 50 µg/mL) in the plates. About 31 ± 11 transconjugants of *C. cellulovorans* 743B were reliably obtained after 1–3 days under strict anaerobic incubation. Thus, the triparental conjugation method proved to be suited for the transfer of various reporter or deletion plasmids into *C. cellulovorans*.

### Establishment of a reporter system to quantify the expression strength of heterologous promoters

The choice of the promoters is important for the balanced expression of genes belonging to the Cas9-based gene editing system. In order to identify suitable promoters, the strength of various promoters in *C. cellulovorans* 743B and *E. coli* was tested. The promoters of *araE* from *Clostridium* *acetobutylicum* (CA_C13399); promoter of *upp* from *Clostridium ljungdahlii* (CLJU_c02320); two ferredoxin *fdx* promoters from *Clostridium sporogenes* (CLSPOx_00425 gene) and *Clostridium saccharobutylicum* (CLSA_c01660 gene) and the thiolase *thl* promoter from *C. acetobutylicum* (CA_C2873 gene) (Table 4) were cloned upstream of the FAST reporter gene in the pMTL83155 vector between the *Not*I and *Nde*I restriction site. The plasmids were transferred into *C. cellulovorans* 743B via triparental conjugation, and the fluorescence signal of the FAST protein was quantified spectrophotometrically. All promoters proved to be active in *C. cellulovorans* during the mid-exponential phase, and their strength was compared by the fluorescence signal generated by the FAST expression plasmids in comparison to the pMTL83155 vector control (fold change compared to negative control, FC_neg_).

**Table 4:** Quantification of heterologous promoters in *C. cellulovorans* 743B

Promoter	Organism of origin	FC_neg_
*P*_*fdx*_	*C. acetobutylicum*	42.9 ± 6.6
*P*_*fdx*_	*C. saccharobutylicum*	5.6 ± 1.4
*P*_*thl*_	*C. acetobutylicum*	4.6 ± 0.7
*P*_*araE*_	*C. acetobutylicum*	9.0 ± 1.3
*P*_*upp*_	*C. ljungdahlii*	3.9 ± 0.4
Vector control	**-**	1.0 ± 0.1

The promoter regions of various genes derived from heterologous Clostridia as indicated, were inserted upstream of the FAST reporter gene in pMTL83155. The results are the average values and standard deviations of at least three biological replicates measured in technical triplicates

The strongest fluorescence signal was obtained for the *fdx* promoter from *C. sporogenes* corresponding to 42.9 ± 6.6 FC_neg_ (Table 4). The *araE* promoter from *C. acetobutylicum* proved to be about 4.8-fold less active in *C. cellulovorans*, while the *fdx* promoter from *C. saccharobutylicum* and the *thl* promoter of *C. acetobutylicum* yielded about 7.7- and 9.3-fold weaker fluorescence signals, respectively, than those found with the *C. sporogenes fdx* promoter. Of the promoters tested, the *upp* promoter from of *C. ljungdahlii* showed the weakest expression level in *C. cellulovorans*, being 11-fold less active than the *C. sporogenes fdx* promoter.

**Fig. 2:**
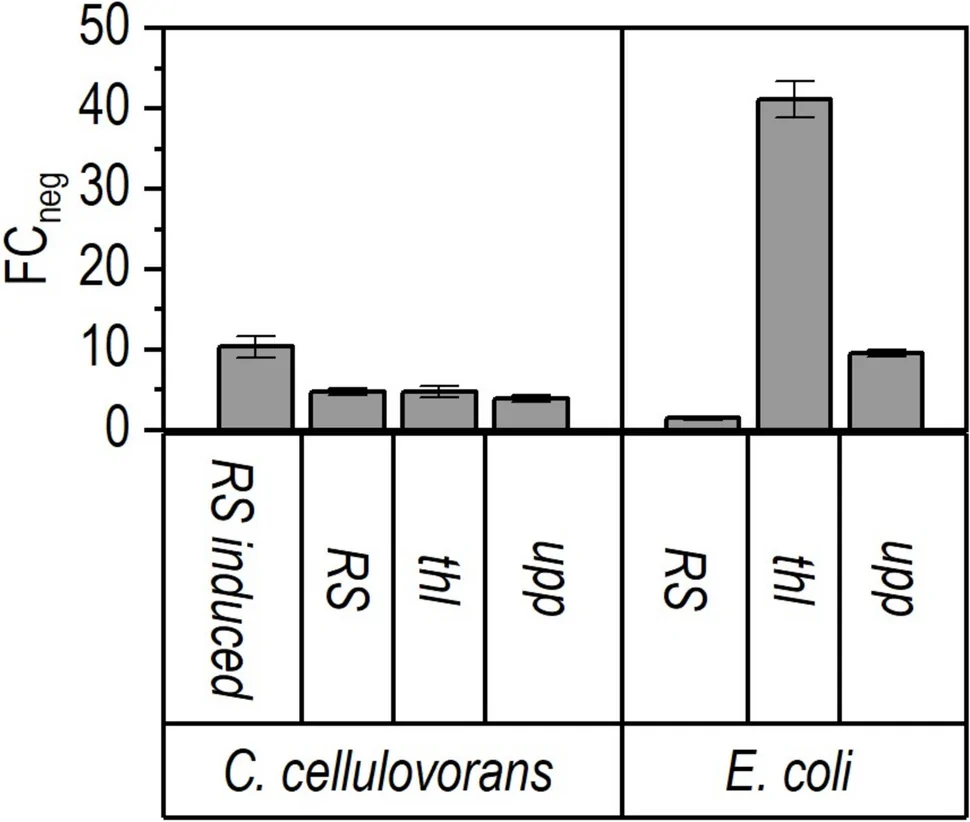
Characterization of the theophylline-dependent riboswitch regulated promoter for Cas9 expression. The promoter activities were quantified using the FAST reporter gene and are given in fold change (FC_neg_) in comparison to the negative control for better comparison between the species. The value 1 corresponds to the vector control using pMTL83155. The riboswitch in the on-state was induced with 5 mM theophylline (RS induced), while the basal activity of the riboswitch was measured in the absence of the inducer (RS)

The theophylline-dependent translational riboswitch E* equipped with the P_*fdx-*_Rb promoter derived from the RiboCas plasmid series was evaluated for the controlled Cas9 expression (Topp et al. 2010; Cañadas et al. 2019). The region between the Shine-Dalgarno sequence and the start codon was modified with an *Nde*I site in order to enable cloning into pMTL83155 using *Not*I and *Nde*I, resulting in the vector pMTL83155-RS. The activity level was then tested with the FAST reporter assay in *C. cellulovorans* (Fig. 2).

The maximal fluorescence signal resulting from pMTL83155-RS in *C. cellulovorans* (10.4 ± 1.3 FC_neg_) was obtained by induction with 5 mM theophylline. A basal activity of the riboswitch of about 4.8 ± 0.4 FC_neg_ in the uninduced state was observed.

Expression of the anti-CRISPR protein AcrIIA4, which binds and inhibits the Cas9, with a suitable promoter can help to lower this potential background activity of Cas9 in the uninduced state. The *upp* and *thl* promoters showed comparable levels of activity to the uninduced activity of the riboswitch in *C. cellulovorans* (4.6 ± 0.7 FC_neg_ and 3.9 ± 0.4 FC_neg_), respectively. The strength of these promoters was also tested in *E. coli*. Here, the activity of the *thl* and *upp* promoter clearly exceeds the basal activity of the riboswitch (off-state, 1.5 ± 0.2 FC_neg_; *thl*, 41.1 ± 2.3 FC_neg_ and *upp*, 9.6 ± 0.4 FC_neg_) as determined by the FAST assay in *E. coli*. To ensure moderate expression of AcrIIA4 in both *C. cellulovorans* and *E. coli*, the *upp* promoter was selected for the construction of the Cas9-based deletion plasmid.

### Controlling Cas9 activity by combining riboswitches for Cas9 expression and repression of basal activity by AcrIIA4

**Fig. 3:**
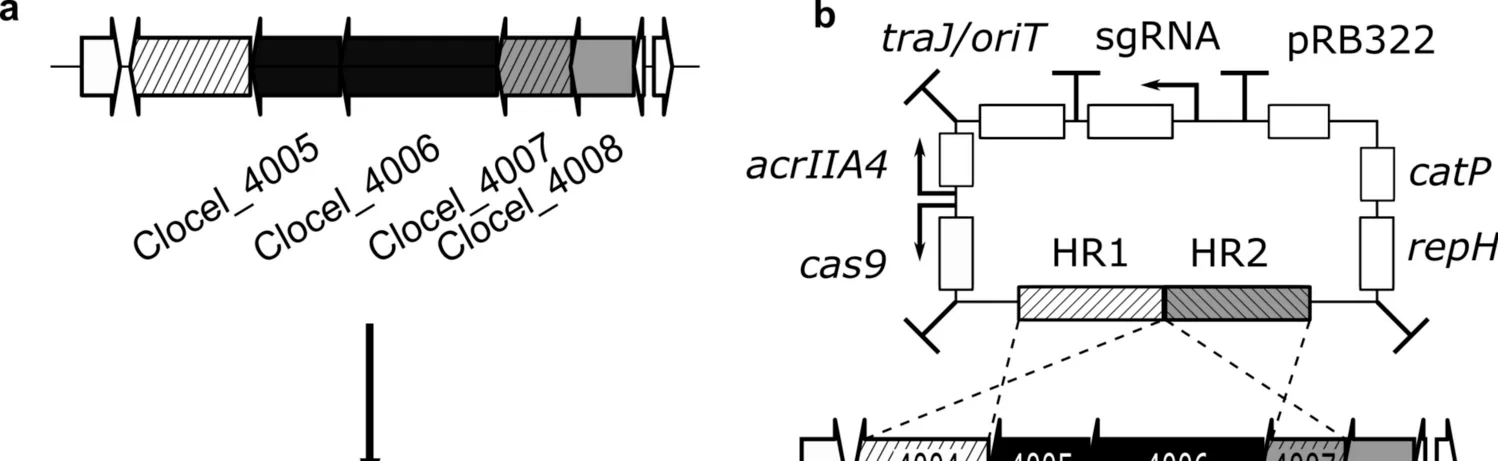
Deletion of the restriction endonucleases Clocel_4005 and Clocel_4006. **a** The WT chromosomal locus of the putative type II RM system containing two putative restriction endonucleases, Clocel_4005 and Clocel_4006, and two methyltransferases, Clocel_4007 and Clocel_4008 (above), and the restriction deficient mutant ΔtypII (below). **b** Schematic representation of the deletion plasmid and the deletion of the restriction endonucleases Clocel_4005 and Clocel_4006 by homologous recombination with 700-bp long homology repair arms (HR) of the plasmid pAcrCas_delClocel4005&4006_ori3 located up- and downstream of the target region (striped region). The target site of the Cas9 is indicated with an arrow

The vectors pAcrCas_delClocel4005&4006_ori3 and pAcrCas_delClocel4005&4006_ori8 were constructed based on the low copy number shuttle plasmid pChN, which was previously developed in our group (Huang et al. 2018). It allows robust conjugative transfer rates in different Clostridia. The vectors were designed as deletion vectors that were to be used to delete the ORFs encoding two type-II RMS endonucleases and enable better genetic accessibility of *C. cellulovorans*. This will facilitate further metabolic engineering using our Cas9-based deletion system (Fig. 3a).

The pChN backbone vector was amplified using primers AS75 and AS74 and ligated with the *fdx* terminator, the Gram-positive replicon, and the resistance cassette from either pMTL83151 (Heap et al. 2009) using *Pme*I and *Nhe*I to generate pAS5. Homologous regions (HR) of 700 bp upstream and downstream of the target gene (primers AS76-AS79) were inserted at the *Stu*I restriction recognition site of pAS5 using NEBuilder to generate pAS5-delClocel_4005&4006. In the following steps, the plasmid pAS5-delClocel_4005&4006 was linearized by PCR (primers ES12 and ES13) to exchange the *codBA* gene by the *cas9* (primers S29 and ES17) while the *codBA* terminator remained. The promoter in front of the *cas9* gene was substituted by the theophylline-responding riboswitch (primers AS112 and AS113), and the *upp* promoter (primers AS114 and AS111) was inserted upstream to control the *acrIIA4* gene terminated by terminator derived from the CD0164 gene of *Clostridium difficile* (synthetic construct). This *acrIIA4* cassette was equipped with two *Not*I sites that allow quick removal of the cassette when needed. Finally, the sgRNA under the control of an *araE* promoter was flanked by a CA-C2406-2407 terminator and an *araE* terminator (synthetic construct) was inserted between the Gram-negative replicon and the *oriT* (primers ES10 and ES11). A suitable seed sequence for the sgRNA to induce the strand break within the Clocel_4005 gene was identified using CRISPy-web2 with default settings (Blin et al. 2016). The seed exchange was performed by oligonucleotides bearing the desired seed region in a PCR reaction (primers SalI_sgRNA_seed2_fw and SalI_sgRNA_rv) and *Sac*I restriction prior to ligation to achieve circularization of the DNA (Fig. 3b).

**Fig. 4:**
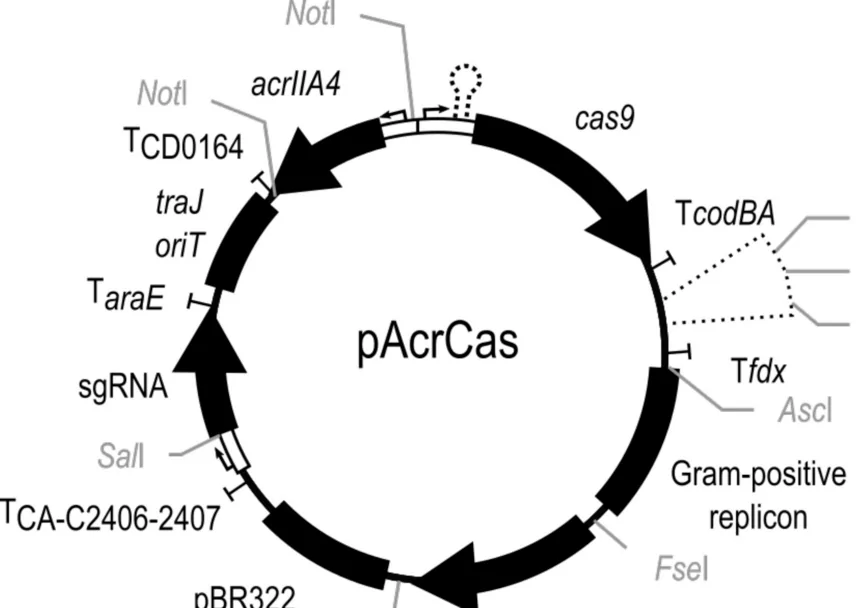
Vector map of deletion vector pAcrCas. The acrIIA4 gene is under the control of an *upp* promoter of *C. ljungdahlii*, and the Cas9 is under the control of a theophylline riboswitch. The HR regions can be inserted using the *Bsa*I or *Eco*47I restriction sites. The Gram-positive replicon and the resistance cassette module are derived from the pMTL8000 series. The sgRNA under the control of an *araE* promoter of *C. acetobutylicum* can be exchanged via PCR reaction, subsequent *Sal*I digestion, and self-religation

Additionally, plasmid pArcCas was generated to facilitate further genetic work (Fig. 4). Here, two *Bsa*I recognition sites suitable for one-pot GoldenGate cloning, and one *Eco47*III recognition site were inserted for vector linearization to facilitate the insertion of other HR sequences (primers AS122_Eco47I_BsaI_Tfdx_fw and AS128_BsaI_MCS_rv) resulting in a flexible vector system.

### Chromosomal deletion of the putative type II restriction endonucleases Clocel_4005 and Clocel_4006

The chromosomal deletion of the ORFs encoding the putative type II restriction endonucleases Clocel_4005 and Clocel_4006 was carried out to overcome the restriction barrier against incoming recombinant DNA in *C. cellulovorans* 743B (Fig. 3). The deletion plasmid pAcrCas_delClocel4005&4006 was in vivo methylated using *E. coli* NEB10β containing placORMI and was transferred into the *C. cellulovorans* 743B WT strain by triparental conjugation resulting in four transformants. The transconjugants were grown overnight in a liquid medium and plated onto plates supplemented with 10 µg/mL thiamphenicol and 5 mM theophylline. The genotype of 24 colonies was analyzed by colony PCR using primers Del3 and Del4, which confirmed the deletion of the Clocel_4005&4006 ORFs with a yield of around 50%.

**Fig. 5:**
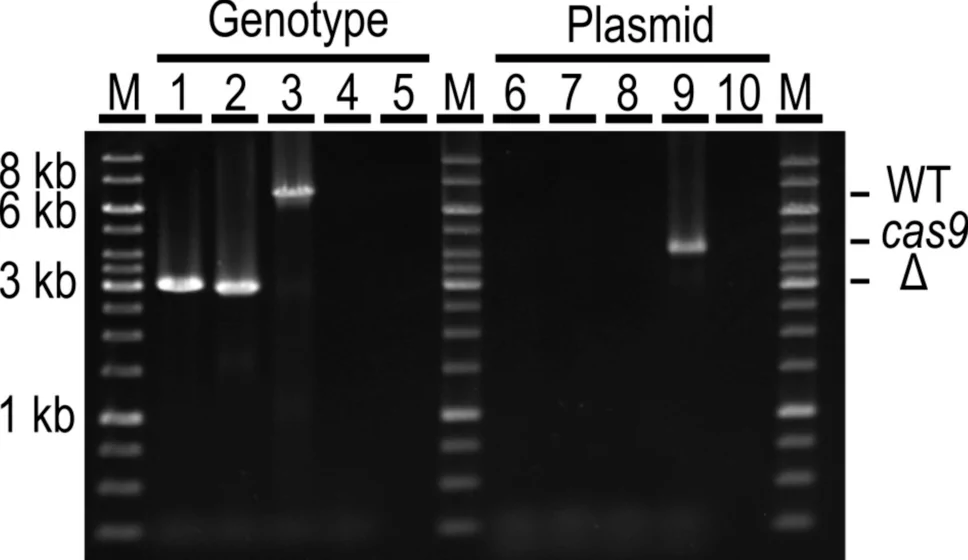
Confirmation of the Clocel_4005&4006 deletion and plasmid curing using PCR amplification. (1–5) Check PCR with primer Del3&Del4 to confirm Clocel_4005&4006 deletion (3 kb) or WT (7 kb). (6–10) Check PCR with primer S29&S30 for *cas9* (4.1 kb). (1, 2, 6, 7) *C. cellulovorans* ΔtypII; (3, 8) *C. cellulovorans* WT; (4, 9) pAcrCas_delClocel4005&4006 plasmid; (5, 10) water

Plasmid-cured deletion strains were obtained by repeated cultivation (1 to 3 passages) in an antibiotic-free medium. The chromosomal deletion and plasmid loss were verified for two thiamphenicol-sensitive colonies using a PCR on the *cas9* gene (primers S29 and S30) and the deletion region (primers Del3 and Del4) (Fig. 5). Additionally, the colonies were inoculated into liquid 2 T medium with and without thiamphenicol to prove their sensitivity toward the antibiotic (data not shown). Finally, the deletion of Clocel_4005 and Clocel_4006 was confirmed by sequencing.

### Effect on the lack of the type II RMS on conjugation efficiency in *C. cellulovorans *ΔtypII

For the *C. cellulovorans* 743B WT, we obtained an average of 1.48 × 10^−7^ transconjugants per WT recipient cell when the shuttle plasmid was in vivo methylated with placORMI bearing Clocel_4007 and Clocel_4008 associated with the type II RMS (Table 5). Conjugation occurs in rare cases without previous methylation (in total merely 1 clone in four independent experiments).

**Table 5:** Conjugation efficiency of *C. cellulovorans* 743B WT and the ΔtypII RMS deletion mutant

Recipient	Unmethylated donor DNA	In vivo methylated donor DNA
WT	2.47 ± 4.23 × 10^−8^	1.48 ± 0.74 × 10^−7^
ΔtypII	2.74 ± 2.18 × 10^−6^	1.08 ± 0.48 × 10^−6^

The efficiency values are given as the number of conjugants per recipient cell with a standard deviation of three independent experiments with pMTL83151 as shuttle plasmid

The conjugation efficiency for the deletion strain *C. cellulovorans* ΔtypII was improved to 2.74 × 10^−6^ transconjugants per ΔtypII recipient cell, meaning that the transfer of plasmids without prior in vivo methylation was about one order of magnitude higher than the wildtype strain conjugated with in vivo methylated shuttle plasmids.

## Discussion

### Conjugation and in vivo methylation

In this study, a great improvement of DNA transfer into *C. cellulovorans* was achieved by the deletion of a putative restriction system using a newly established Cas9-based gene editing system. This study represents the first report of a functional conjugation protocol for *C. cellulovorans,* which allows efficient conjugation and chromosome editing in *C. cellulovorans*.

In contrast to transformation by electroporation, which has been described previously (Yang et al. 2015), triparental conjugation allowed obtaining transconjugants in the WT strain at a low frequency even without any protection of the transferred DNA from restriction in the recipient. The advantage of triparental conjugation compared to a diparental conjugation is that it allows direct usage of well-established laboratory *E. coli* strains for conjugation, such as *E. coli* NEB10 that do not contain *tra* genes, simply by adding a helper strain such as *E. coli* CA434. During conjugation, plasmids are often less affected by the restriction modification system of the acceptor strain because they are transferred in the single-stranded form during the conjugative transfer (Jennert et al. 2000; Des Purdy et al. 2002). The transconjugation efficiency in *C. cellulovorans* was improved by adding a bicarbonate buffer to the mating plates. This could counteract the accumulation of fermentation products of *C. cellulovorans* and *E. coli* during the mating process, which could negatively affect the growth of *C. cellulovorans* due to its low tolerance toward low pH values (Sleat et al. 1984). In vivo methylation was done from a weakly induced regulated promoter, because in our experience with other organisms, a comparatively weak expression of the methylases is already effective, but circumvents detrimental effects for the host cell.

### Quantification of heterologous promoters using FAST

The characterization of several heterologous promoters in *C. cellulovorans* yielded a selection of promoters with different strengths as a toolbox to choose from for the rational design of a Cas9-based gene editing system for this bacterium. The *fdx* promoter of *C. sporogenes* and the *thl* promoter of *C. acetobutylicum* are frequently used in Clostridia due to their strong expression (Heap et al. 2009; Tummala et al. 1999; Huang et al. 2016). Similarly, the *fdx* promoter of *C. sporogenes* also displayed high activity in *C. cellulovorans*. In contrast, the data of this study indicated only a low activity of the *thl* promoter in *C. cellulovorans*. The data for *C. cellulovorans* was confirmed for the exponential and stationary phase, which is important to note because the expression level of the *thl* promoter is linked to the growth phase in other Clostridia like *C. acetobutylicum* (Tummala et al. 1999; Girbal et al. 2003).

For the development of Cas9-based deletion systems, the choice of a promoter with an adequate strength for expression of the sgRNA is important (McAllister et al. 2017; Wang et al. 2017). The *araE* promoter was successfully used for sgRNA expression in different Clostridia (Huang et al. 2016; Cañadas et al. 2019) and was also functional for *C. cellulovorans*.

The least active promoter in *C. cellulovorans* in this study was the *upp* promoter from *C. ljungdahlii*. We used the *upp* promoter for the expression of the AcrIIA4 protein, resulting in a functional Cas9-based gene editing system. Besides, weak promoters also are useful for membrane protein expression or counter-selection strategies in genetic engineering. For example, an *upp* promoter was used by us previously to establish *codBA*-dependent counterselection for markerless chromosomal editing in acetic acid bacteria (Kostner et al. 2013).

### Chromosomal editing in *C. cellulovorans*

Previous reports of genetic engineering of *C. cellulovorans* have reported the application of plasmid-based expression systems (Yang et al. 2015), gene silencing by dCas9, or gene disruption by ClostTron/TargeTron technology (Wen et al. 2017). We now provide a tool for markerless chromosomal deletions for *C. cellulovorans* by using a Cas9-based counterselection strategy including the avoidance of undesired effects of Cas9 by expression of an anti-CRISPR protein.

The power of gene editing using Cas9 was reported for different Clostridia with editing efficiencies between 12.5 and 100%. The success rate is highly dependent on the chromosomal target site but is also influenced by the length of the HR template (Li et al. 2016). The editing efficiency of 50% obtained in this study is consistent with efficiencies reported for other Clostridia (Nagaraju et al. 2016; Huang et al. 2016; McAllister et al. 2017; Mertaoja et al. 2021). The poor efficiency of the introduction of DNA has been a major hindrance to strain development in *C. cellulovorans* and also impaired the number of transconjugants obtained with the Cas9 vectors. Anyhow, this result is in agreement with the low number of transformants reported for *C. acetobutylicum* (Li et al. 2016; Bruder et al. 2016), *C. ljungdahlii* (Huang et al. 2016), and *C. pasteurianum* (Pyne et al. 2016) due to the high lethality of double strands breaks caused by unspecific endonuclease activity of Cas9 (Xu et al. 2015; Cui et al. 2020; Wang et al. 2016; Nagaraju et al. 2016; Wen et al. 2017). Thus, various strategies have been used to overcome the toxicity of Cas9.

Often, inducible promoters are used to fine-tune the *cas9* expression. Prominent systems included promoters regulated by regulatory proteins responsive to lactose (Wang et al. 2016), xylose (Wilding-Steele et al. 2021), or tetracycline (Marcano-Velazquez et al. 2019; Nagaraju et al. 2016; Wasels et al. 2017, 2020; McAllister et al. 2017), or by artificial riboswitches (Cañadas et al. 2019). The purpose of the regulation is to shut off cas9 expression during plasmid construction in *E. coli* but to induce it in the host organism for gene editing.

The application of a small theophylline-dependent riboswitch (Topp et al. 2010; Cañadas et al. 2019) allowed to keep the overall plasmid size below 11 kb but resulted in a basal activity of Cas9 in *C. cellulovorans*.

Previous reports indicate that a minimal level of the *cas9* expression is sufficient to cause cytotoxicity (Wasels et al. 2020). Recently, the anti-CRISPR protein AcrIIA4 was described to bind to Cas9 in a 1:1 ratio, preventing the binding of sgRNA and thus inhibiting the activity of Cas9 (Shin et al. 2017). The strategy to use anti-CRISPR as an antagonist for Cas9 was first successful for a two-plasmid system in *C. acetobutylicum* (Wasels et al. 2020). In this study, the AcrIIA4 protein was expressed from a weak promoter in a single-plasmid system together with a riboswitch-controlled *cas9*. The idea was to inhibit weak Cas9 activity resulting from basal expression of *cas9,* but not to hamper Cas9 activity in an induced state. The disadvantage of this approach is a larger plasmid that must be introduced, which requires a more efficient conjugation system.

### Deletion of the putative type II restriction-modification system

The disruption of type II restriction barriers was shown to improve the genetic accessibility and facilitate the engineering of other Clostridia (Cui et al. 2012; Croux et al. 2016), so we decided to delete the putative type II RM system of *C. cellulovorans* 743B, which was previously described to represent a barrier for DNA introduction by electroporation (Yang et al. 2016). The putative operon of the type II RM system is unusual because it encodes two methyltransferases Clocel_4008 and Clocel_4007, and two restriction enzymes Clocel_4006 and Clocel_4005. A similar structure was described for the lactococcal plasmid pNP40, where all four proteins of the LlaJI operon are necessary for phage resistance (O’Driscoll et al. 2004). As LlaJI is expressed in a temperature-dependent manner (O’Driscoll et al. 2004), we hypothesized that small temperature changes might also influence the type II RM system of *C. cellulovorans* 743B and explain the variable results during independent transconjugation experiments. Detailed analysis of the lactococcal methyltransferases M1.LlaJI and M2.LlaJI reveals different recognition sequences that can control the transcription of the LlaJI operon by the asymmetric arrangement of the recognition sequences in the promoter region (O’Driscoll et al. 2005). In contrast, no regulatory function of the recognition site could yet be identified in *C. cellulovorans*. The restriction unit R1.LlaJI functions as a specificity unit, allowing the heterodimer R1.LlaJI-R2.LlaJI binds to the DNA, while R2.LlaJI is responsible for the DNA cleavage (O’Driscoll et al. 2006). To ensure the lowering of the restriction barrier in *C. cellulovorans*, both putative endonucleases of the type II RM system were selected for deletion to ensure complete inactivation before studying the effect on the conjugation efficiency.

The conjugation efficiency with the *C. cellulovorans* ΔtypII strain as the recipient was tenfold higher when a pMTL83151 plasmid without *C. cellulovorans*-specific methylation was transferred, as compared to the 1 to 4 colonies obtained by conjugation into the WT recipient using a donor strain that carries out in vivo methylation of the plasmid. These experiments show that Clocel_4005 and Clocel_4006 represent an important restriction barrier in *C. cellulovorans* and that their deletion resulted in conjugation efficiencies high enough for much easier genome editing and further exploitation of *C. cellulovorans* for biotechnological applications, since it enables routine usage of large (10 kb) deletion plasmids. This new strain will be the basis for further improvement of genetic accessibility, including further optimization of the Cas9 system or the development of alternative gene editing strategies. Also, it cannot be excluded that there are additional protection systems against foreign DNA in *C. cellulovorans* whose inactivation could further improve the efficiency of the genome and metabolic engineering of this efficient cellulose-utilizing bacterium in the future.

In conclusion, this study resulted in providing tools for markerless metabolic engineering of *C. cellulovorans*, including a protocol for triparental conjugation, a library of functional heterologous promoters, and a Cas9-based system for chromosomal editing that will facilitate further work with this organism.

## Data Availability

The vector maps and data of this study are available from the corresponding author upon request.
